# Assessing the mental wellbeing of next generation general practitioners: a cross-sectional survey

**DOI:** 10.3399/bjgpopen19X101671

**Published:** 2019-10-16

**Authors:** Fanny Lindemann, Zsofia Rozsnyai, Brigitta Zumbrunn, Julia Laukenmann, Regula Kronenberg, Sven Streit

**Affiliations:** 1 MD Candidate, Institute of Primary Health Care (BIHAM), University of Bern, Bern, Switzerland; 2 Deputy Head of Career Development, Institute of Primary Health Care (BIHAM), University of Bern, Bern, Switzerland; 3 Resident, Department of General Internal Medicine, Bern, Inselspital, Bern University Hospital, Switzerland; 4 Resident, Primary Care Practice, Lenzerheide, Switzerland; 5 Resident, Department of General Internal Medicine, Lucerne, Cantonal Hospital Lucerne, Switzerland; 6 Head of Career Development, Institute of Primary Health Care (BIHAM), University of Bern, Bern, Switzerland

**Keywords:** general practitioners, mental wellbeing, mental health, training, residency, resilience, surveys and questionnaires

## Abstract

**Background:**

Future and practising GPs encounter various stressors, which can potentially impair mental wellbeing and develop into mental illnesses.

**Aim:**

To assess mental wellbeing of young and future GPs by their level of training.

**Design & setting:**

A cross-sectional anonymous survey of members of the Swiss Young General Practitioners Association (JHaS) was undertaken.

**Method:**

Basic characteristics and the current mental wellbeing were assessed using the Warwick-Edinburgh Mental Wellbeing Scale (WEMWBS). Specific stressors that can influence wellbeing were focused on. Participants were asked for ideas on how to improve wellbeing via open questions.

**Results:**

Response rate was 57% (*n* = 503). Mean value for mental wellbeing (WEMWBS) was 52.4 (maximum 70, standard deviation [SD] 7.2). Residents had a significantly lower level of mental health (51.0, SD 7.6) compared with GPs (54.2, SD 6.2). Overall, stress level was reported as high or very high by almost half of participants (49%). Forty-five per cent indicated a lack of private time; the highest proportion was among residents. Fifteen per cent (20% among residents) were at risk of burnout. Most frequent stressors were administrative tasks, high workload, and work demands. Support requests included improvement of work–life balance and reduction of administrative workload.

**Conclusion:**

Residents had the lowest mental wellbeing, at a stress level similarly high to that of GPs. They most often indicated not having enough time for a private life and were most at risk of burnout. Improvement suggestions should be implemented to maintain mental health of young and future GPs. Particular attention should be paid to GPs in training, as owing to their reduced mental health, they may benefit most.

## How this fits in

Against the background of a growing shortage of GPs, it is important to secure the mental wellbeing of the next generation GPs. This study assessed mental wellbeing in students, residents, and early- career GPs. All participants were member of JHaS, with >1100 people committed to becoming GPs. It was found that mental wellbeing during GP training was the lowest, and was most often affected by work–life imbalances and burnout symptoms. Participants called for a reduction of administrative work and workload to improve their mental wellbeing in the long term.

## Introduction

Physicians face a variety of stressful challenges including high workload,^1,2^ and difficulties balancing work and home life owing to long working hours and overtime.^3^ Such work-related factors could reduce mental wellbeing, and ultimately lead to burnout or mental illness such as depression^4–6^ or anxiety disorders. If physicians are unwell or emotionally exhausted, serious consequences might follow; for example, greater willingness to leave their profession,^7,8^ and even risk to patients' safety owing to potentially increased medical errors.^9,10^ Against the background of a growing shortage of GPs,^11^ it is alarming to see that physicians at frontline care are at highest risk of burnout.^12^

This GP shortage may be partially alleviated by a burgeoning interest in family medicine.^13,14^ Young and future Swiss GPs have concrete ideas regarding their future practice,^13^ and the introduction of an attractive postgraduate training programme noticeably increased their interest in general medicine.^14^

These are important steps towards countering another shortage of GPs. Nevertheless, little is known about the mental health of the young generation of Swiss GPs. In order to prevent burnout, or the manifestation of mental illnesses, there is a need to focus on GPs' wellbeing and how it can be maintained in the long term.

The aim of the study was to assess the mental wellbeing of future and young Swiss GPs. The study set out to understand which work factors put a particular strain on them, and extract these according to their level of training. Further, the study let the next generation of GPs have their say by asking them to formulate concrete support measures that can help promote their mental health.

## Method

### Setting and participants

Since Swiss GPs in training are not listed in a national register, JHaS was surveyed as the second best option for the target population. JHaS is an organisation founded in 2006 with >1100 members, including medical students and residents committed to becoming GPs, as well as GPs up to 5 years in practice (Switzerland has about 7000–8000 GPs in total). Participants’ intention to become a GP was ascertained when they entered the study, in order to ensure only those who planned to become GPs were included in analyses. Those who were not committed (4%) were excluded.

### Survey and measurements

An online questionnaire with four sections was developed:

#### Mental health

Section one was administered to all participants, which assessed age, sex, whether or not they had children, current workload (half-days per week), and current level of mental wellbeing based on the 14-item WEMWBS.^15^ The WEMWBS tests subjectively perceived wellbeing and psychological function during the last 2-week period on a 5-point Likert scale (14–70 points, higher = better mental health). The scale was not invented as a screening method for mental illness and there is no cut-off value.^15^ WEMWBS has been used in various studies and validated in several languages and settings.^16–18^ A user licence was received to include WEMWBS in the study (submission ID: 482086307).

#### Stress levels

Section two was for all participants and covered the topics of stress and its consequences. Participants were asked to rate their current stress level (5-point Likert scale) and indicate: if they had ever suffered from burnout in the past, how often they lacked time for their private life, and how often they thought about giving up their profession (5-point Likert scale). Like the WEMWBS, these questions covered the previous 2-week period. Since this short period could have skewed results by, for example, holidays or illness, it was specified that the questions should refer to the last 2 weeks the participants worked. To estimate the current burnout risk, a common applied single-item, self-defined burnout measure was used,^19,20^ which shows a strong correlation with the Maslach Burnout Inventory exhaustion subscale^21^ and has been used in other studies.^20,22,23^

#### Training level-specific stressors

In section three training level-specific questions were asked about current working conditions that could affect wellbeing. Residents and GPs were asked what proportion of time they spent on administrative work (5-point Likert scale), and to indicate how often they felt burdened by different stressors during their last 2 weeks of work. Lists varied by level of training and were based on stressors frequently mentioned in literature.^1,4,7,24,25^

#### Improvement suggestions

The survey was concluded with section 4, in which participants were given the opportunity to describe the support they needed to increase or maintain mental health (open question).

#### Participation

In order to increase participation rates, the following steps were undertaken: (1) the study was announced in a newsletter before members received access to the online survey; (2) a raffle was offered for three book vouchers worth about €80 each; and (3) three reminders were sent to non-responders. The survey was completely anonymous. It was distributed in January 2019 in German and French.

### Sample size considerations

Murray *et al* ^26^ detected a difference of mental wellbeing using the same WEMWBS score in the youngest (49.7, SD 7.8) compared with the oldest age group (52.1, SD 8.2). To detect a similar difference with 80% power and level significance of 5%, it was calculated that a sample size of about 352 participants was needed.

### Data management

Categorical data were described as proportions; data were analysed across the three strata (level of education) and compared by χ^2^ test. Missing data occurred in just 7.9% and were handled in a restricted analysis. To determine the participants' current risk of burnout, five possible responses were dichotomised into two categories (symptoms of burnout versus no symptoms of burnout), as in other studies.^23,27^ Questions were also dichotomised about current stress level, not enough time for a private life, and thoughts about leaving the profession after visual checks of the distributions using histograms. For the WEMWBS, means and 95% confidence intervals (CIs) were calculated using a crude regression model stratified by level of education. The results of the WEMWBS were analysed for each of the 14 questions separately.

### Statistical analysis

To test for an association between level of training and mental wellbeing (WEMWBS), a multivariable linear regression model was used. In a causal modelling, the study aimed to estimate causal associations between the exposure (level of training) and the outcome (mental health, that is, WEMWBS score). The authors, a priori, chose sex as confounder. The crude effects of level of training and mental health were then looked for in order to assess the degree of confounding for each covariate compared with the crude model. Covariates were kept that confounded the association of level of training and mental health by at least 10%. Potential confounders were: age, language, civil status, having children, part-time work, current stress level, time for a private life, leaving profession, and current and past burnout. Covariates with strong pair-wise correlation (>0.60) were reduced to avoid multicollinearity. The crude or adjusted coefficients and 95% CIs were calculated.

A qualitative approach was undertaken to analysing the free-text answers to open questions about suggested improvements. The first author coded the answers and built categories in consultation with the last author; disagreements were rare, and were resolved by consensus. The percentage frequency was calculated with which the topics were mentioned and it was decided to present those which were frequently mentioned in tabular form (lower limit at 5%). Open answers, which could not be assigned to any category in terms of content, were given their own code.

A two-sided *P* value of 0.05 was considered to be statistically significant. All data were analysed in Stata (version 15.1).

## Results

### Participants

The questionnaire was emailed to all 1115 JHaS members. A total of 918 (82%) opened the email; it was assumed that 197 (17%) of members had not seen or received the email. From those 918, 523 (57%) participated in the survey. Twenty participants (4%) were excluded because they stated they did not intend to become GPs. The answers of the remaining 503 participants were analysed.

Most participants were women (*n* = 352 , 75%), mean age was 33.6 (SD 5.7); 42% were married and 36% were in a relationship; and 42% had children (Table 1). The proportion of parents was highest among GPs (30%), closely followed by residents (27%). The largest segment of responders was in training as residents (48%).

**Table 1.** Baseline characteristics of participants, stratified by level of education

Characteristics	Overall *n* = 503	Medical students *n* = 46 (9.8%)	Residents *n* = 228 (48.3%)	GPs^a^ *n* = 196 (41.7%)	*P* value
**Sex, *n* (%)**					
Female	352 (75.1)	43 (93.5)	168 (74.0)	141 (71.9)	0.009
**Mean age, years (SD)**	33.6 (5.7)	25.5 (2.0)	31.7 (3.8)	38.1 (4.3)	<0.001
**Language, *n* (%)**					0.016
German	430 (91.5)	43 (93.5)	200 (87.2)	187 (95.4)	
French	40 (8.5)	3 (6.5)	28 (12.3)	9 (4.6)	
**Civil status, *n* (%)**					<0.001
Single	104 (22.2)	19 (41.3)	63 (27.6)	22 (11.3)	
With partner	167 (35.6)	27 (58.7)	92 (40.4)	48 (24.6)	
Married	198 (42.2)	0 (0)	73 (32.0)	125 (64.1)	
**Children, *n* (%)**	198 (42.3)	0 (0)	62 (27.2)	136 (70.1)	<0.001
**Part-time work, *n* (%)**	246 (52.8)	14 (32.6)^b^	67 (29.4)^b^	165 (84.6)	<0.001

33 (6.5%) participants did not specify if they were students, residents, or GPs and were therefore only analysed in the overall column.

^a^GPs early in their career. ^b^Total *n* = 466, due to missing data.

### Mental health

**Figure 1.**
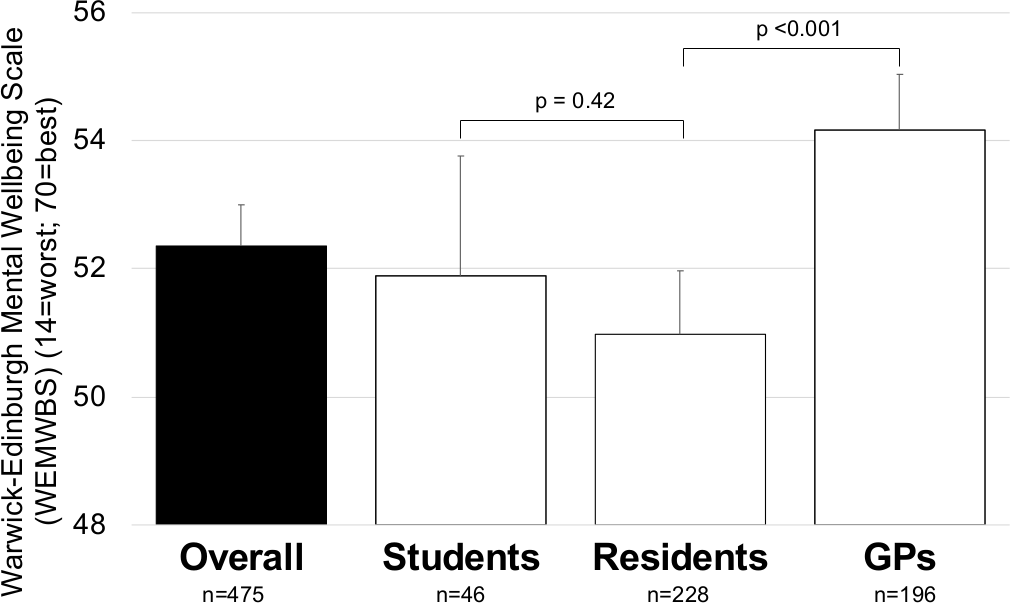
Crude comparison of mental wellbeing in young and future GPs, and by level of education (total *n* = 475, due to missing data *n* = 28)

Figure 1 describes the mean value of all participants' mental wellbeing on the WEMWBS of 52.4 points (95% CI = 51.7 to 53.0). Looking at sub-scales of WEMWBS, it was found 85% of all participants were ‘often’ or ‘all the time’ interested in other people, but only 47% had energy to spare, and only 29% felt relaxed. GPs had a significantly higher feelings of wellbeing (54.2, 95% CI = 53.3 to 55.0) than residents (51.0, 95% CI = 50.0 to 52.0, *P*<0.001); students' wellbeing fell between the groups (51.9, 95% CI = 50.0 to 53.8). Fewer than 50% of participants reported often or very often having energy to spare (47%) or feeling relaxed (29%).

### Stress levels

Almost half of the participants reported high or very high stress levels (49%) and too little time for their private lives (45%); with the risk being much higher among residents (56%; *P*<0.001, Table 2). Few (8%) thought about leaving the profession often or very often; but most of these were residents (11%). Fifteen per cent were at risk of burnout; the highest proportion (20%) was among residents and 17% of all responders had experienced burnout at some point during their career with the highest distribution among residents (17%) and GPs (19%).

**Table 2.** Stress and sequelae of stress of future and early-career GPs, stratified by level of education

Characteristics of stress, *n* (%)	Overall *n* = 470^a^	Medical students *n* = 46 (9.8%)	Residents *n* = 228 (48.3%)	GPs^b^ *n* = 196 (41.7%)	*P* value
**Stress level, high or very high^c^**	229 (48.7)	19 (41.3)	114 (50.0)	96 (49.0)	0.56
**Not enough private time, often or very often^c^**	210 (44.7)	15 (32.6)	128 (56.1)	67 (34.2)	<0.001
**Thinking of leaving job, often or very often^c^**	37 (7.9)	1 (2.2)	24 (10.5)	12 (6.1)	0.08
**Current risk of burnout^d^**	70 (14.9)	4 (8.7)	45 (19.7)	21 (10.7)	0.016
**Ever experienced burnout or exhaustion or depression**	79 (16.9)	3 (6.5)	39 (17.3)	37 (18.9)	0.13

^a^Total *n* = 470 due to missing data. ^b^GPs early in their career. ^c^Asked in respect of the last 2 weeks; recorded on a 5-item Likert scale (very often–never), dichotomised in two groups (often or very often versus sometimes or rarely or never). ^d^Measured in five categories and dichotomised based on Edwards *et al.* ^23^

### Training-specific stressors

**Figure 2.**
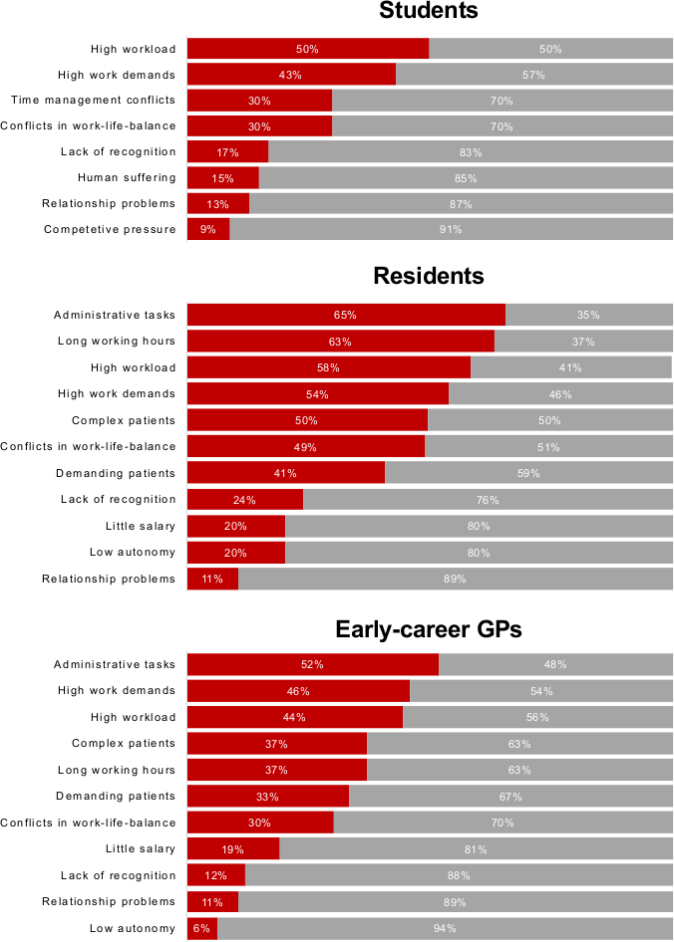
Stress factors of students, residents, and early-career GPs sorted by frequency. Participants responding (very) often are shown as red bars, the rest as grey bars.

Both residents and GPs found coping with administrative tasks particularly burdensome (frequently or very frequently burdensome for 65% of residents and 52% of GPs, Figure 2). Residents indicated long working hours were almost as burdensome (65%), followed by high workload (58%), and work demands (54%). GPs indicated high work demands (46%) and high workload (44%) were particularly onerous. Students rated high workload as the most burdensome (50%), followed by high work demands (43%), conflicts in time management (30%), and conflicts in work–life balance (30%).

### Modelling level of education and mental health

In a multivariate regression analysis (Table 3), different factors associated with mental wellbeing were extracted: women had lower levels of wellbeing than men (by 1.7 units, *P* = 0.022). Having children was associated with increased wellbeing (by 2.0 units, *P* = 0.008); working part-time hours was associated with lower level of wellbeing (by 1.6 units, *P* = 0.062). The feeling of not having enough time for a private life was associated with a significantly lower level of wellbeing (by 4.5 units, *P*<0.001).

**Table 3.** Final multivariable regression model: the effect of level of education on mental wellbeing (*n* = 463). Adjusted for all covariates in the table

Participant characteristic	Adjusted difference in mental wellbeing (95% CI)	*P* value
**Level of education**		
Student	0.5 (-1.8 to 2.7)	0.69
Resident	reference	
Early-career GP	2.2 (0.7 to 3.8)	0.005
**Sex**		
Female	-1.7 (-3.1 to -0.2)	0.022
Male	reference	
**Children**		
Yes	2.0 (0.5 to 3.5)	0.008
No	reference	
**Part-time work**		
Yes	-1.6 (-3.2 to 0.1)	0.062
No	reference	
**Not enough private time**		
Often or very often	-4.5 (-5.8 to -3.2)	<0.001
Sometimes, rare, never	reference	

Reading example: having children (compared with not having children) means a higher score in WEMWBS of 2.0 points (95% CI = 0.5 to 3.5 points).

Sample size here *n* = 463 is smaller due to missing data in 8% of the sample.

### Improvement suggestions

Participants suggestions to improve their mental health are summarised in Table 4. About one fifth of participants (19%) thought it particularly important to improve work–life balance and reduce administrative activities. They wanted a family-friendly working environment, acceptance and promotion of part-time work, and better childcare. To reduce administrative tasks, participants suggested non-medical staff could support them, as could electronic medical records and efficient computer programs. Shorter working hours and adherence to breaks were recommended by 11% of participants. Responders wanted to avoid overtime and observe breaks. Other topics mentioned most frequently included improvements in education and teaching, regular mentoring, more feedback, and close supervision.

**Table 4.** Support requests for optimisation and long-term preservation of mental wellbeing

Frequency	Main theme	Examples mentioned
19%	Improving work–life balance	family-friendly working environment acceptance and promotion of part-time work less shift-work improving childcare
19%	Reduction of administrative tasks	assistance from non-medical staff use of electronic medical records efficient computer programs
11%	Shorter working hours and adherence to breaks	no overtime adherence to breaks
10%	Improvement of education and training	regular mentoring at all training levels improving feedback culture, dealing with mistakes close supervision improving teaching
10%	Workload reduction	more time for individual patient reduction of high work demands measures against the lack of family doctors
6%	Good team atmosphere and collegial exchange	regular exchange among colleagues organised case discussions
6%	Recognition	by superiors, colleagues, and environment
5%	Salary adjustment	possibility of calculating work in the absence of patients (preparation and follow-up time)

## Discussion

### Summary

This was the first study assessing mental health across different career periods of young and future GPs. Residents reported significantly lower mental wellbeing compared with early-career GPs. Lack of private time and risk of burnout were highest in residents compared with students and GPs. High stress levels (49%) and intention to leave the profession (8%) were equally reported across career periods. The most common stressors among GPs and residents were administrative tasks, high workload, and high work demands. Students thought high workload and high work demands were most burdensome. Strongest inverse associations with mental wellbeing were, independently, having not enough time (4.5 units lower), being female (1.7 units lower), and potentially working part-time (1.6 units lower). Having children was associated with higher reported mental health (2.0 units higher). Participants recommended measures to improve their work–life balance and to reduce administrative workload.

### Strengths and limitations

The strengths of the study are the novelty of surveying young and future GPs to compare mental health in different career points, and choosing to survey JHaS members on mental wellbeing since there is no national registry of young and future GPs. Switzerland has about 8000 practising GPs and >1100 of the next generation of GPs were invited. Thus, to the authors' best knowledge, a nationally representative sample of next generation GPs was surveyed. Although women were likely to be overrepresented in the study, with a 75% share compared with men, >50% of medical students in Switzerland are female, therefore, it is believed this sample is valuable to study. The study was sufficiently powered to detect similar differences as in comparable studies or settings.^26^ It was also helpful to let participants suggest what should change to improve their mental health.

It is acknowledged that the study had limitations: firstly, selection bias can be substantial if those with lower mental health tended to participate less. However, similar mental health among first responders and responders answering late was observed. Secondly, there is risk of social desirability bias, which can potentially be lowered through anonymity of responses.^28^ Thirdly, although statistical significance was found, the clinical relevance of, for example, 2 units lower in WEMWBS can be questioned; the effect sizes were, however, equal to those by Murray *et al*.^26^ The authors argue that factors such as female sex, working part-time hours, and lack of private time can accumulate (in that case to 7.6 units).

### Comparison with existing literature

Overall, this sample of young and future Swiss GPs reported higher mental health (52.4) compared with UK GPs (50.2), teachers (47.2), or the general population of Northern Ireland (50.8).^26^ It was found that wellbeing in residents was significantly lower compared with young GPs (*P*<0.001); residents most often stated a lack of private time (*P*<0.001) and were at highest risk of burnout, when compared with GPs and medical students. These results align with findings from other studies that identified the training period as the phase most characterised by distress and difficulty.^4,29,30^ Despite high stress levels, GPs had the highest mental wellbeing among the subgroups, and higher compared with the GP sample surveyed in the cross-sectional study of Murray *et al* (50.2 versus 54.2 in the present sample). These results show that after completion of the training period, wellbeing increases among practising Swiss GPs and is higher compared with a similar GP sample in the UK. In contrast to Murray *et al*'s sample, in which women had potentially higher mental health than men, mental wellbeing of women in the present sample was lower compared with men (+1.7 to –1.7 units in this sample).

It was found that working part-time was associated with lower mental health (1.6 units). One reason for this may be the partial lack of acceptance of part-time work, as many of the participants in the study mentioned. Although having children was associated with increased wellbeing (2.0 units), participants complained of insufficient childcare. Consequently, mothers working part-time may experience a double burden, and still have to cope with a similarly high workload, resulting in less private time.

Administrative tasks, long working hours, conflicts in work–life balance, and heavy workload were rated as most burdensome by both residents (65%) and GPs (52%). Other studies highlighted the same factors as main sources of physicians' stress.^7,25,30,31^

The combination of high perceived stress, and rather infrequent thoughts of leaving the profession aligns with Hayes *et al*’s results: hospital doctors in Ireland complained of poor work–life balance and high work stress, but were nevertheless motivated to pursue their profession.^30^ Other recent studies^13,14^ also show that young physicians are highly motivated to practise as GPs.

### Implications for research and practice

Intervention studies to reduce stress and avoid burnout in physicians are rare,^32^ but a randomised controlled study showed that regular debriefing sessions had a positive effect and participants considered them to be helpful support measures.^31^ Studies focusing on stress management methods to support the individual are valuable and may help doctors to deal better with stress.^33^ However, the study findings support the claim that physician wellbeing and burnout are structural problems within health organisations, as several other studies have emphasised.^12,31,34,35^ More prospective randomised intervention studies are needed to address the problem at its source.

Young and future GPs are, overall, dedicated to their profession, despite operating in conditions of high stress. They have clear needs that might feasibly be met to ensure their mental health over the long term. Residents in GP training might benefit most from intervention measures since their stress levels are particularly high and they have lower mental health compared with students and early-career GPs.
